# Medical and Philosophical News

**Published:** 1781-03

**Authors:** 


Hair, bones, and even complete teeth have ſometimes been found in the female ovarium. Facts of this kind, though perhaps of no great practical utility, cannot fail of proving intereſting to the anatomical reader; we ſhall therefore here mention ſome ſingular appearances that lately prefented themfelves to a celebrated profeffor of anatomy in London, on diffecting a female fubject at his lectures.—Upon opening the cavity of the abdomen, the inteftines, bladder, and uterus were feen forming one difeafed flefhy-like mafs; the Fallopian tubes were indurated and difeafed, and in a word, the whole of thefe parts were fo much confufed as not to be eafily diftinguifhable. But the moft extraordinary circumftance appeared on opening into the rectum; for a little above the fphincter were two tumours, if we may fo call them, placed one immediately above the other; the largeft of the two was about two inches in length, an inch in breadth, and of proportional thicknefs; the other was much fmaller. Both were covered by the inner tunic of the inteftine reflected over them, and were each of them fufpended as it were by a pedicle compofed of feveral fibrillæ. On cutting into their fubftance there were found a quantity of hair, and feveral perfect teeth, incifores, bicufpides, and molares.—Nothing is known of the hiftory of the fubject, but from certain appearances fhe was judged to be about thirteen years of age, and the hymen was obferved to be perfect.

